# Experimental test of birdcall detection by autonomous recorder units and by human observers using broadcast

**DOI:** 10.1002/ece3.4775

**Published:** 2019-02-10

**Authors:** Isabel Castro, Alberto De Rosa, Nirosha Priyadarshani, Leanne Bradbury, Stephen Marsland

**Affiliations:** ^1^ Wildlife and Ecology Group Massey University Palmerston North New Zealand; ^2^ School of Mathematics and Statistics Victoria University of Wellington Wellington New Zealand; ^3^ Terrace End, Palmerston North New Zealand

**Keywords:** acoustic surveys, bioacoustics, bird surveys, forest birds, point counts, recording technique

## Abstract

1. Autonomous recording units are now routinely used to monitor birdsong, starting to supplement and potentially replace human listening methods. However, to date there has been very little systematic comparison of human and machine detection ability.
2. We present an experiment based on broadcast calls of nocturnal New Zealand birds in an area of natural forest. The soundscape was monitored by both novice and experienced humans performing a call count, and autonomous recording units.
3. We match records of when calls were broadcast with detections by both humans and machines, and construct a hierarchical generalized linear model of the binary variable of correct detection or not, with a set of covariates about the call (distance, sound direction, relative altitude, and line of sight) and about the listener (age, experience, and gender).
4. The results show that machines and humans have similar listening ability. Humans are more homogeneous in their recording of sounds, and this was not affected by their individual experience or characteristics. Humans were affected by trial and location, in particular one of the stations located in a small but deep valley. Despite recorders being affected significantly more than people by distance, altitude, and line of sight, their overall detection probability was higher. The specific location of recorders seems to be the most important factor determining what they record, and we suggest that for best results more than one recorder (or at least, microphone) is needed at each station to ensure all bird sounds of interest are captured.

## 1 INTRODUCTION

There is a need for effective bird monitoring methods to assess species presence, abundance, evaluate the consequences of current species management‐for‐conservation practices, and to provide an indication of overall balance in a given biome (Dawson & Efford, 2009; Digby, Towsey, Bell, & Teal, 2013; Towsey, Planitz, Nantes, Wimmer, & Roe, 2012; Vielliard, 2000). Birdsong is often used to detect, monitor, and quantify species because it works even when the individuals are out of sight. Humans are capable of identifying birds aurally with reasonable accuracy: The average person can recognize birdcalls in their backyard, while experts can identify hundreds of bird species by their song alone. It is therefore not surprising that birdcall surveys are a common method of assessing populations of birds and conservation managers have turned to some of these methods to monitor species for conservation purposes.

Surveys carried out by humans have been shown to have issues arising from varying ability to detect and identify species (Alldredge, Simons, & Pollock, 2007; Diefenbach, Brauning, & Mattice, 2003; Emlen & DeJong, 1992; Sauer, Peterjohn, & Link, 1994; Simons, Alldredge, Pollock, & Wettroth, 2007), changes in behavior of birds due to human presence (Bye, Robel, & Kemp, 2001; Hutto & Young, 2003; McShea & Rappole, 1997), and misclassification of species (Farmer, Leonard, & Horn, 2012; Sauer et al., 1994), to varying hearing ability of observers (Ramsey & Scott, 1981). Additionally, human surveys can be logistically challenging and costly. Furthermore, most of the methods used for measuring bird populations are not well suited and/or are unaffordable for species in low numbers (Sutherland, Newton, & Green, 2004).

Advances in technology have seen an increase in the use of autonomous recording units (ARUs) for monitoring of bird populations. This technology has been recognized for having the potential to overcome some of the human issues, and for having some extra advantages. For example, ARUs are less likely to affect birds’ behavior, and their sampling can be scheduled in advance and carried out at selected times of day and night over long periods (Telfer & Farr, 1993; Hobson, Rempel, Greenwood, Turnbull, & Wilgenburg, 2002; Rempel, Francis, Robinson, & Campbell, 2013), allowing these devices to be placed in remote locations and minimizing temporal biases in sound recording. Further, ARUs produce archival records that allow the listener to replay and verify identifications of species (or ask other listeners to do so) and can be deployed by people with limited bird knowledge.

Given that it is likely that ARU recordings will increasingly replace, or at least supplement, human listening, the key question is to what extent the recordings are comparable to human hearing. This is particularly important as one of the first steps to make this technology useful to conservation and/or research is to develop protocols, which requires knowledge of the strengths and limitations of the ARUs for capturing sounds under a range of conditions. This knowledge is also important for the development of methods of analysis of the data collected via ARUs, and to judge the validity of abundance estimates obtained from ARUs surveys.

Since the beginning of the 2000s, a number of studies have compared ARUs and humans during the common bird survey types (Salamon et al., 2016; Table 1). Most of these studies use simultaneous recording by ARUs and observers in natural settings. The challenge in analyzing these data is the lack of a gold standard: The machine recording is compared to the paper annotation of the human observers. Since the lack of human consistency is one of the drivers for ARU adoption, this seems problematic at best. In addition, detection ranges differ between these survey methods. The ability of humans to move their heads and therefore capture sounds from several directions means that even if recorders and humans get overall similar results in surveys, the way they achieve this would be different. Therefore, the protocols to be used by each method should be calibrated to achieve comparable results.

**Table 1:** Field studies examining the differences between acoustic recorders and human observers, and details of equipment used for recording, habitat type, observers, performance measures, and what the author's considered pluses and minuses of Autonomous Recording Units when compared to human observers

References	Equipment	Location and vegetation	Observers	Method	Performance measures	Pluses	Minuses
Haselmayer and Quinn (2000)	Portable cassette recorder (Marantz) and a highly directional microphone	Peru (seasonal flooding areas, canopy height ranging from 35 to 40 m, and closed canopy)	Not specified	Point counts. Simultaneous recording	Two environmental factors (estimated richness and presence of noisy species) and two attributes of species (abundance and foraging height) on estimates of species richness	1. ARUs detected more species per station than point counts by human observers 2. Allow repeated listening	1. Mean overall number of species detected was lower for ARUs (missing rare species and those that vocalize rarely)
Hobson et al. (2002)	Digital recorder, one omnidirectional microphone, and two directional microphones	Canada (Boreal mixed‐wood Forest)	One per site	Point counts. Simultaneous recording	Species abundance and number	1. Extended sampling efforts 2. Increased opportunity to replicate monitoring activities 3. Control for observer variability 4. Recordings can be interpreted by a single or multiple trained experts as necessary 5. Allows the standardization of field data through time. 6. Archived record of point counts 7. Non‐expert field staff can collect recordings 8. Data from recordings could be completed following the field season when experts are less in demand and less expensive to hire or by a single trained researcher conducting the scientific work at no extra cost 9. Attenuation in song from digital recordings can be measured and distance estimated by comparison with empirical data 10. Field data can be readily backed up to a hard drive on a personal computer	1. Possible inability to control or evaluate the distance over which recordings were made and 2. Determining accurate relative abundance estimates for each species
Acevedo & Villanueva‐Rivera (2006)	Manual recorder (Marantz), one omnidirectional microphone, and a custom‐made controller	Puerto Rico (mangrove/brackish‐forested wetland	Not stated	Point counts. Simultaneous recording	Number of birds (day) and number of amphibians (night)	1. Better quantity and quality of data 2. Permanent record of a census. 3. 24 hr/day data collection 4. The possibility of automated species identification	1. Lack of density estimates 2. Detection is limited to calling individuals, whereas traditional point counts include visual observations
Celis‐Murillo, Deppe, and Allen (2009)	Manual recorder fixed in a point and 4 omnidirectional microphones	California, USA (Riparian Forest and Southern Willow Scrub)	One expert	Point counts. Simultaneous recording	Estimate bird species abundance, richness, and composition	1. Higher detection probabilities of individual birds and earlier detection 2. Better at detecting rare species of conservation concern 3. No interobserver difference 4. more reliable estimates of detection probability and abundance 5. Produce permanent records of surveys, 6. Resolve problems associated with limited availability of expert field observers 7. Elimination of interobserver and minimization of intra‐observer error by using a single interpreter in the laboratory 8. Costs of equipment can be offset by using non‐experts in the field and experts in the lab	1. Future research needs to develop standards and protocols for estimating absolute sampling area are required before acoustic recording methods can be appropriately incorporated into studies comparing bird density among habitats, seasons, geographic locations, or species. 2. Composition of species different to point count 3. May require a greater effort, particularly more time
Hutto and Stutzman (2009)	Cornell recorders	Montana, USA (green mixed‐conifer forest, burned mixed‐conifer forest, and mixed riparian cottonwood bottomland)	One experienced; One inexperienced observers	Point counts. Simultaneous recording	Detection of birds and species	1. Superior, for identifying species by song 2. Recordings can be reviewed later. 3. Greater interobserver consistency 4. Having a permanent archive of the sounds 5. Ideal for nocturnal surveys because distracting ambient sounds are minimal at night and danger to human observers is probably at its maximum 6. Determine whether a particular species is present in a fairly restricted area 7. detection of rare species	1. Lower mean number of species detected 2. Lower detections of individual birds 3. More time‐consuming and expensive 4. data loss due to equipment malfunction
McGuire, Johnston, Robertson, and Kleindorfer (2011)	Korg MR−1000 recorder +Telinga Twin Science parabolic or Sennheiser MKE 80R shotgun microphone	Eyre and Yorke Peninsulas, Australia (whipbird habitat)	Not stated	Playback of local Western Whipbird songs; and (2) automated recording stations	Number of surveys sites where whipbirds were recorded (presence)	No difference in detectability using the two methods 2. Can be set up by community groups and subsequently scored by someone with the skills to recognize Western Whipbird songs 3. A permanent record of the survey remains	1. Playback method was less labor intensive than the use of automated recording stations (which required hours of postcollection data analysis)
Celis‐Murillo, Deppe, and Ward (2012)	Manual recorder fixed in a point and 4 omnidirectional microphones	Mexico‐Yucatan Peninsula (coastal dune scrub, mangrove, low‐stature deciduous thorn forest, early and late successional medium‐stature semi evergreen forest, and grazed pastures)	One observer	Point counts. Simultaneous recording.	Species richness and composition, and detection probabilities of 15 rare, moderately common, and common tropical bird species	1. The two methods provided comparable estimates of richness and composition, and vegetation type did not affect the relative performance of the methods 2 can be used to survey remote areas without the need for trained field surveyors	1. Cannot detect birds/species that are not vocalizing 2. Detection probabilities for different species were influenced by survey method either independently or interactively with vegetation type 3. Analyses of recordings can be expensive and time‐consuming
Venier, Holmes, Holborn, McIlwrick, and Brown (2012)	SONGMETER SM1 (two omnidirectional microphones) and E3A Bio‐Acoustic Monitor Kit	Canada (Boreal forest).	Unclear but one to two per site.	Point counts. Simultaneous recording.	Richness (no. of species) and abundance, and 2) richness based on number of visits	1. E3A more species than SM1 and human observers 2. advantageous in situations where the number of experienced observers is limited, where access difficult, where multiple samples at the same site are desirable, and where it is desirable to eliminate interobserver, time‐of‐day and time‐of‐season effects	1. SM1 Fewer species and number of birds than E3A and human observers 2. E3M fewer birds than human observers
Tegeler, Morrison, and Szewczak (2012)	Own built devices	California, USA (wet montane meadows)	12 experienced +extra training	Point counts. Same area and period but not simultaneous	Species richness	1. Viable supplement and potential alternative to standard point‐count surveys to conduct large‐scale avian species richness surveys 2. Provided >1,200 hr of data, 1,000 hr more than point‐count survey 3. Monitor continuously and, therefore, sample more intensively than human observers 4. Better estimation of species richness 5. Possible to glean some demographic information from particular call types 6. detection of rare or specific species	1. Failure of recorders 2. Slight but significant less detection of species 3. Recorded audio data cannot readily estimate species abundances because current systems have only limited ability to estimate distances and number of individuals 4. Cannot estimate the proportion of individuals present in the sampling area that are not producing acoustic cues
Digby et al. (2013)	Song Meter SM2.	New Zealand (broadleaf regenerating forest).	2–3 observers.	Point count. Simultaneous recording.	Detection capability, time requirements, areal coverage, and weather condition bias	1. Area coverage comparable with field surveys 2. Spectrogram inspection 30 times more efficient than traditional counts 3. Reduction in temporal and observer bias	1. Less calls recorded 2. Fixed in location thus no further information about birds 3. Greater effect of wind 4. Did not detect increase in calls due to change in ground condition
Zwart, Baker, McGowan, and Whittingham (2014)	SONGMETER SM2+ SM1 (two omnidirectional microphones).	Northumberland, UK (coniferous woodland, heather moorland, and a small amount of deciduous woodland).	Not stated	Line transect. Used ArcGIS to plot locations of nightjars’ line‐transect data. Used nearest recorder within a 500 m radius for each located nightjar registration and for each recorder, and each visit, to get presence/absence data.	Detection of nightjars	1. Recorders were better detecting nightjars 2. Allowed finding the best time to survey nightjars 3. Good for surveys for species that do not vocalize regularly 4. Causes less disturbance than traditional surveys 5. Benefit when surveys are in remote or difficult to access areas, as visits need only be made when deploying and picking up the recorders or replacing the batteries 6. Can be deployed by locals and recordings analyzed by experts	None recorded
Lambert and McDonald (2014)	Note taker (Olympus 8,600‐VN, Australia)	Australia (no vegetation, dry sclerophyll forest and rainforest).	Not stated.	Visual scanning. Simultaneous recording and visual scanning (No aural detection by people).	Detection of birds	1. Provided accurate estimates of population density throughout the range of the visually cryptic bell miner 2. Acoustic method detected more birds. 3. No need for high skill observers 4. Data comparable within and between sampling years 5. Provides permanent data record 6. Inexpensive	None
Borker, Halbert, McKown, Tershy, and Croll (2015)	SongMeter SM2 one omnidirectional microphone.	California, USA (marbled murrelet sites).	Five trained observers.	Audio‐visual scanning. Simultaneous recording.	Mean rate of detections per survey using traditional inland audio–visual surveys with indices measured using ARUs. Measured cumulative likelihood of detecting one murrelet call given successive mornings of acoustic monitoring	1. Greater ability to sample and more economical thus provides ability to expand the number of samples for similar cost to human observer surveys 2. Increase the temporal and spatial scale of sampling and reducing biases 3. Good for surveying remote areas for murrelets 4. Permanent record of surveys 5. Eliminate inter‐ and intra‐observer bias 6. No need for trained observers in the field	1. Fewer (less than half) detections per sampling thus slower to detect small populations 2. Removal of a human observer comes with some statistical and cost advantages, but no microphone will match the ecological insights to be gained from a human observer
Klingbeil and Willig (2015)	Wildlife Acoustics SongMeter Sm2 +) with two omnidirectional microphones	Connecticut, USA (deciduous and coniferous forests).	Not stated.	Point counts. Simultaneous recording and season's surveys	Number of species and individuals	1. Higher species numbers if using season's surveys	1. Detected fewer species and fewer individuals in simultaneous surveys
Alquezar and Machado ( 2015)	SongMeter SM2+	Brazil (Brazilian cerrado and other open vegetation areas)	1 observer	Point counts. Simultaneous recording.	Detection of birds and species	1. No significant differences between the number of species detected by point counts and ARUs 2. Good to be able to keep a record that can be used in future 2. ARUs can extend sampling at the same point at different times of the day increasing the chance of detecting more species that may be silent at the time of a single point count 3. Allows long‐term and standardized acoustic monitoring.	Processing time for recordings (for each 15 min of a sample unit, we took twice this time to analyze it) 2. Depending on the desired analysis, data obtained can be viewed as biased, because not all species had the same chance to be registered 3. No abundance, no data on species density and behavior, no information of species microhabitat preferences
Sidie‐Slettedahl et al. ( 2015)				Robust‐design occupancy models	Detection probabilities	Recording units may be effective for surveying nocturnal secretive marsh birds if investigators correct for differential detectability	1. Reduced detection
Leach, Burwell, Ashton, Jones, and Kitching (2016)	SongMeter SM2+	Queensland, Australia (rainforest)	One expert	Point counts using recordings collected in the field as well as field point counts.	Mean proportions of the total species detected at the sites. Effect of elevation stratification	1.There was significant overlap in the species detected using both methods, but each detected several unique species 2. No differences in the community‐level patterns (elevational stratification and turnover in species composition with increasing elevational distance between sites) 3. The permanent record of a community generated by acoustic recording allows for the re‐analysis of the data using novel techniques in the future	1. Detected less species for an equivalent length of time 2. Need to process the recordings manually 3. The adverse effects that the weather and the ambient acoustic environment can have on recording quality 4. Cost of equipment and 5. Hardware problems 6. Inability to accurately generate abundance or density data
Vold , Handel, and McNew (2017)	Song Meter SM2	Alaska, USA (boreal forest and Arctic tundra)	One experienced	Point count. Simultaneous recording.	1) Numbers of birds and species 2) Effect of distance on detection probabilities 3) test avian guild and habitat influence detection	Pairing of the 2 methods could increase survey efficiency and allow for validation and archival of survey results	1. detected fewer species and fewer individuals
Wilgenburg, Solymos, Kardynal, and Frey ( 2017)	Song Meter SM2+ ARU with a pair of SMX‐II microphones.	Saskatchewan, Canada (boreal forest).	Five observers.	Point count. Simultaneous recording.	Number of species	1. Raw counts derived from both acoustic recordings and human observers were relatively comparable	
Yip et al. ( 2017)	Song Meter SM2, SM3, RiverForks CZM, Zoom H1 handheld recorders	Alberta, Canada (10 road sites, 5 coniferous forests, and 5b deciduous forests).	Not stated	Broadcast experiment comparing how several ARUs and human observers detect sounds at various distances and vegetation types.	Detection/non‐detection. Effect of distance and weather conditions. Detection for sounds of different amplitudes	1. Counts derived from both ARUs and human observers were relatively comparable	In general, humans in the field could detect sounds at greater distances than an ARU although detectability varied depending on species song characteristics

In this study, we compare humans and ARUs by presenting them simultaneously with birdcalls broadcast at various distances and locations. We then look at (a) the effect of distance, sound direction, relative altitude, and line of sight on the capacity of ARUs and people to record bird sounds, and (b) the effect of age, experience, and gender on the ability of observers to hear bird sounds. We used the calls of three of New Zealand's nocturnal species: two kiwi species (*Apteryx owenii*, little spotted and *A. mantelli*, brown) and an owl, the ruru (*Ninox novaezelandiae*). Kiwi is a flightless nocturnal ground insectivorous bird endemic to New Zealand, while the ruru is a small forest owl from Australasia.

Based on sound theory (Forrest, 1994), we predicted that: (a) Calls broadcasted from speakers in locations relatively lower than listening stations would be captured by recorders and humans while those broadcast from higher sites would not, as sound would travel above the recorders/people; (b) speakers located in line of sight of autonomous recorders/human observers would be heard better and there would be less obstruction of the sound waves; (c) low‐frequency calls would be recorded more/better than high‐frequency calls as the latter attenuate more in the forest environment; and (d) shorter distances between speaker and autonomous recorder/human observer would result in better recordings.

## 2 MATERIALS AND METHODS

### 2.1 Experimental design

**Figure 1:**
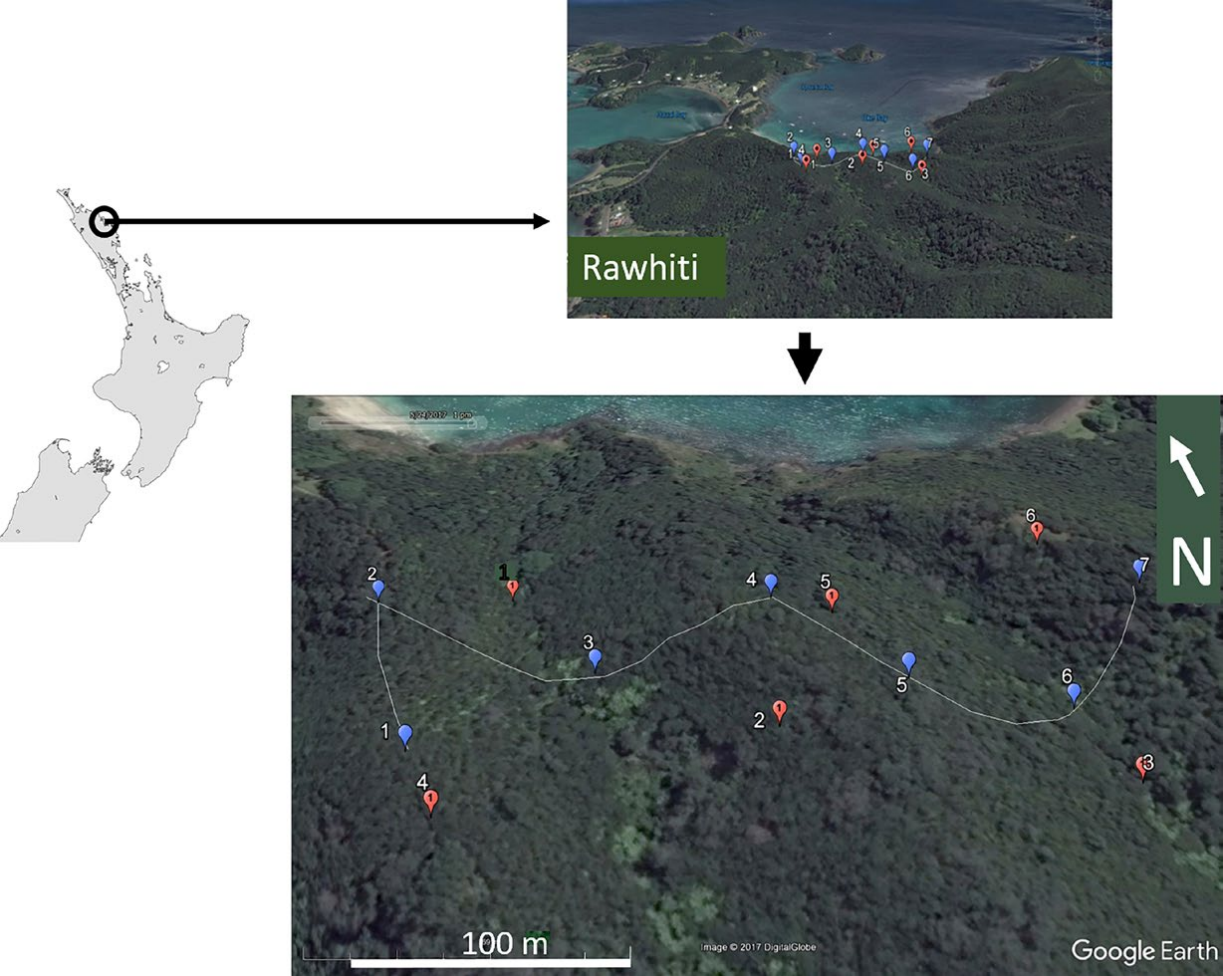
Overview of the location of the experimental site in New Zealand showing the Rawhiti settlement, and the listening stations (blue markers) and broadcasting sites (red markers). Listening stations 3 and 6 were located in valleys; 1, 4, and 7 on hill tops; and 5 and 2 half way up a hill. Broadcasting sites 1 and 3 were located in valleys, 6 and 4 on hill tops, and 2 and 5 on the side of a hill

The experiment took place at Rawhiti, Northland, New Zealand (35.2330°S, 174.2606°E). It consisted of broadcasting prerecorded bird sounds from six broadcasting sites to be recorded by both human observers and ARUs located at seven different listening stations (Figure 1), allowing direct comparison between them. Each human observer carried out the listening exercise at all seven listening stations, resulting in seven trials (Table 2). This enabled us to compare the effects of location without the confounding factors of differences between human observers.

**Table 2:** Observer details and order in which s/he visited the stations to record broadcasted sounds

Observer	Expertise rank (1–4)	5mbc (yr)	KCS (yr)	Other Survey	Age (yr)	Gender	Station order						
1	2	0	10	3	66	Female	1	3	5	7	6	4	2
2	4	0	0	0	28	Male	1	3	5	7	6	4	2
3	3	0	3	1	61	Female	2	1	3	5	7	6	4
4	2	2	4	0	42	Male	3	5	7	6	4	2	1
5	1	1	7	0	73	Female	3	5	7	6	4	2	1
7	0	0	0	0	47	Female	4	2	1	3	5	7	6
6	3	0	4	0	74	Male	4	2	1	3	5	7	6
8	3	0	0	3	54	Female	5	7	6	4	2	1	3
9	0	0	0	1	40	Female	5	7	6	4	2	1	3
10	4	0	0	0	25	Male	6	4	2	1	3	5	7
11	1	0	1	3	45	Male	7	6	4	2	1	3	5
12	2	0	0	0	30	Female	7	6	4	2	1	3	5
13	3	0	0	0	37	Female	6	4	2	1	3	5	7

Note

Expertise rank was self‐assessed using the following categories: 1 = knows most NZ species sounds well; 2 = knows most NZ forest species sounds well including rare birds; 3 = knows a variety of common NZ species sounds well; 4 = knows only a few common species sounds well. 5mbc: Five‐minute bird counts; KCS: kiwi call survey.

Human observers were initially deployed to their first listening station. Each trial then followed the same format: Based on a sound signal (a shotgun blast), a series of bird calls were played from six broadcasting stations. At the end of the broadcast, another shotgun blast informed human observers of the end of the trial. The observers then had 10 min to move to their next listening station, and the next trial commenced. A double shot was fired at the end of the experiment to indicate the time to return to base.

### 2.2 Broadcasts

The six broadcast sites were unknown to human observers, but the observers visited the seven listening stations during the day, prior to the experiment, to become familiar with their location along the track (Figure 1). Experimenters, with their broadcast equipment, were deployed to their locations before the human observers started the experiment to prevent observers knowing the locations of the broadcasts. Speakers were activated by experimenters at fixed times after the start of each trial (gunshot signal). For practical reasons, we used five different speaker combinations for broadcasts: three FoxPro models (Wildfire, FX5, and Firestorm): two Marantz 660 recorders coupled to a Saul Mineroff Portable Field Speaker (SME‐AFS), and a Sony PCM‐M10 recorder coupled with a SME‐AFS. However, prior to the experiment, all the speakers were adjusted to generate the same sound pressure level for a given birdcall.

**Figure 2:**
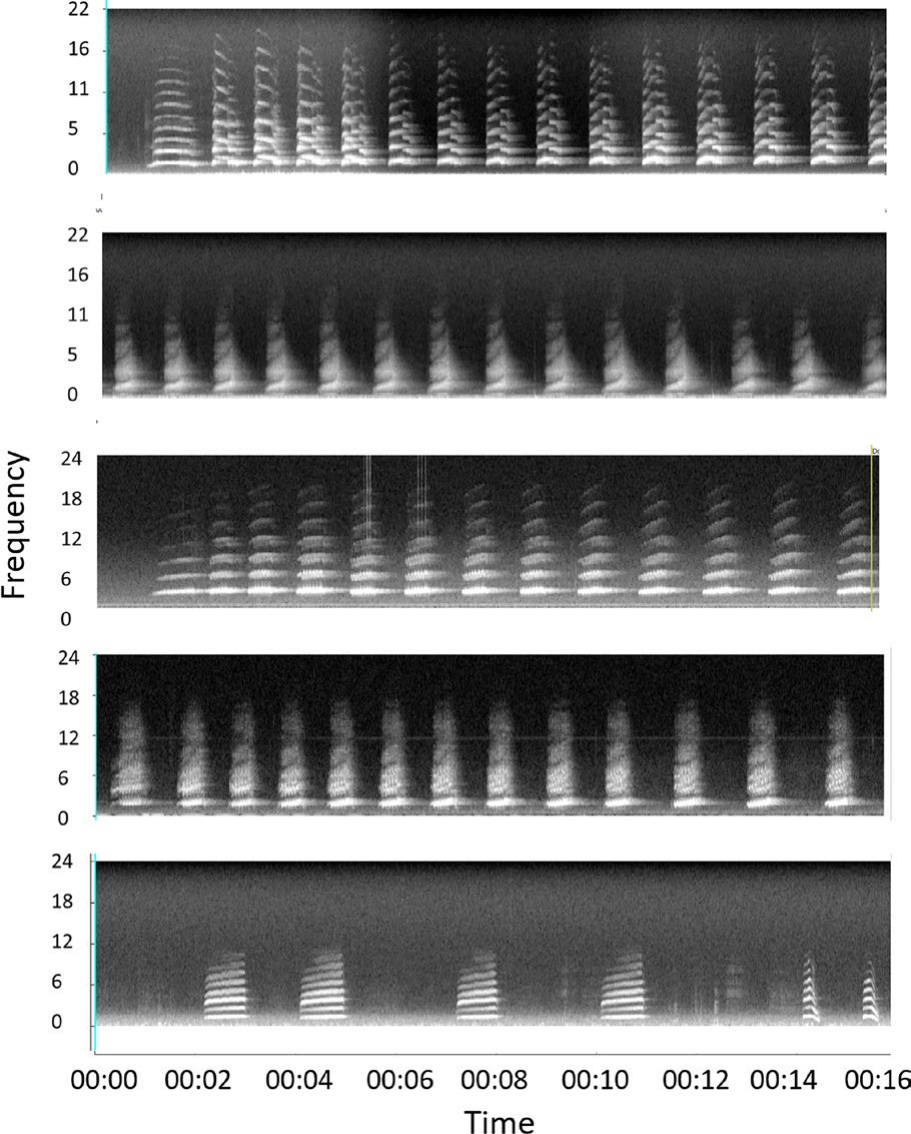
Spectrograms of calls used in this experiment. From top to bottom: brown kiwi male, brown kiwi female, little spotted kiwi male, little spotted kiwi female, and ruru

Broadcasts from different speakers were not supposed to overlap and we expected that observers in most cases could hear sound from several of the speakers (i.e., if they were close to more than one speaker). In practice, some experimenters started the speakers slightly earlier or slightly late and thus some overlap of songs occurred. Each speaker broadcast the calls of three nocturnal birds known to the observers: two species of kiwi, which were not known to exist in the area, and ruru, which exist in low density (Table 3). For kiwi, we used one male and one female call for each of the two species, and for ruru, we used a combination of *trill* and *weow* calls (Brighten, 2015) resulting in five calls being broadcast (Figure 2).

**Table 3:** Species and call sequences used in the Rawhiti Acoustic Experiment

Sequence speaker 1	Sequence speaker 2	Sequence speaker 3	Sequence speaker 4	Sequence speaker 5	Sequence speaker 6
BK female	BK female	LSK female	BK male	BK male	LSK female
LSK female	Ruru	BK female	BK female	LSK female	Ruru
LSK male	BK male	LSK male	Ruru	BK female	BK male
Ruru	LSK male	Ruru	LSK male	Ruru	BK female
BK male	LSK female	BK male	LSK female	LSK male	LSK male

Note

BK: brown kiwi: *Apteryx mantelli*; LSK: little spotted kiwi; *Apteryx owenii*; ruru/morepork, *Ninox novaeseelandiae*.

Previous work indicated differences in transmission of bird sounds between day and night (Priyadarshani, Castro, Marsland, 2018) and so the experiment was conducted between 21:00 and 23:30, which is in the time range where the selected species naturally call. Calls were broadcast at natural volume (Section 2.4 below). Each birdcall sequence was 88 s (1.47 min) long, and therefore, the total amount of hearing time was 7.33 min for each sequence. Each speaker played the songs in a different predefined random order to prevent observers from predicting bird order (Table 3); this was particularly important because the calls remained the same for the entire experiment. The order in which the speakers broadcast the calls was also randomized (Table 4) to prevent observers from predicting where sounds would come from. All speakers broadcast north and were located on the ground facing upwards at 45 degrees to simulate a kiwi calling from the forest floor (I. Castro, pers. obs.).

**Table 4:** Order in which speakers broadcasted song during the Rawhiti Acoustic Experiment

Trial 1	Trial 2	Trial 3	Trial 4	Trial 5	Trial 6	Trial 7
1	6	5	1	2	3	6
6	2	4	3	5	5	5
4	4	2	5	4	4	4
2	3	1	6	1	1	2
5	5	6	4	6	6	3
3	1	3	2	3	2	1

Note

Trials were separated by a 10‐min period, while observers moved from one station to another.

### 2.3 Human observers

Two observers with different level of expertise were located 2–4 m apart at each of the seven listening stations (Figure 1). The two observers were out of sight of each other to prevent them from copying from each other, and to ensure that they were independent in their listening. Each recording station had an autonomous acoustic recorder mounted on a tree at head height above the human observer. The 14 ARUs were units created by the Department of Conservation Electronics Laboratory, Wellington (electronics@doc.govt.nz) recording at 32 kHz. These omnidirectional recorders using 4 x wm61a electret microphones in parallel with a foam “pop filter” and custom‐made low noise pre‐amplifier with a DSP anti‐aliasing filter, −35 dB ±4 dB sensitivity, and 50 Hz to 16 kHz frequency response. The ARUs were programmed to record between 20:30 and 00:00 hours and were on site before the human observers arrived at their stations. Human observers were asked to perform a call survey using data sheets (Appendix 1) similar to those used for kiwi call surveys in New Zealand, which requests details of the species, time of calling, direction (measured using a compass), and distance (estimated by observer from experience). Prior to the experiment commencing, each observer completed a small survey to gather information about their competence, and personal variables that could affect their performance in the experiment (Appendix 2). They were also encouraged to listen to recordings of the species they were going to survey as a training exercise.

### 2.4 Processing of song for broadcast

Each bird call was chosen from high‐quality recordings of the species (Figure 2). The files were denoised using wavelets (Priyadarshani, Marsland, Castro, & Punchihewa, 2016). The selected birdcalls were listened to by IC who is experienced in working with the chosen species in the field. Each song was broadcast to IC who indicated when the volume of the song sounded as if the bird was calling next to her. Once these levels were decided, the songs were concatenated using *Praat* (http://www.fon.hum.uva.nl/praat/) and a tone marker was added at the beginning of the sequence. This way all songs in the recording were at the estimated correct volume when compared to each other.

The broadcasting volume from each speaker was adjusted based on the volume of the initial tone until it was the same for all speakers. One speaker was used to broadcast the song, and all other speakers were calibrated using a sound meter (Digitech QM1592 Professional Sound Level Meter) following manufacturer instructions: The sound meter was placed 20 cm from the ground on a tripod and 1.5 m from the speaker looking directly toward the speaker. Using this method, the volume for the tone ranged between 61 and 63 dB; for brown kiwi female between 75 and 79 dB; brown kiwi male between 79 and 87 dB; little spotted kiwi male between 77 and 81 dB; little spotted kiwi female between 76 and 82 dB; and ruru between 77 and 79 dB (Table 5).

**Table 5:** Average ± standard deviation (*SD*) broadcast decibels for each sound used

Sound	Recorder number (Db)									
	5		6		3		4		1	
	Av.	*SD*	Av.	*SD*	Av.	*SD*	Av.	*SD*	Av.	*SD*
Tone	61.96	7.37	63.38	7.38	62.71	7.14	63.61	6.36	63.08	7.34
BKF	75.83	4.43	75.99	4.50	78.14	6.05	79.72	6.86	78.73	5.91
BKM	79.69	5.95	80.64	6.66	82.62	7.09	89.21	9.64	87.78	11.39
LSKF	76.53	4.87	78.03	4.97	81.51	7.61	82.12	7.90	82.14	7.38
LSKM	77.31	4.10	78.85	4.65	81.95	5.93	77.40	4.29	79.96	5.20
Ruru	77.93	8.33	77.11	8.39	77.95	8.27	77.49	8.23	79.93	8.96

Note

BKF: brown kiwi female; BKM: brown kiwi male; LSKF: little spotted kiwi female; LSKM: little spotted kiwi male. Db: decibels.

### 2.5 Analyses

#### 2.5.1 Data from the recorders

Sound recordings were stored as wav‐files with a 32 kHz sampling rate and 16 bit data depth. We used AviaNZ version 1.0 for the visualization and analyses of sounds (AviaNZ team, Massey University, 2017) using a 256‐sample Hann window. As a first step, a recording from one of the stations was scanned in AviaNZ for the shotgun sounds that defined the beginning and end of broadcast trials. All sounds were annotated for the whole experiment for a single recorder (with the help of other recorders when the calls were not registered or faded). Then, this was used as a template to annotate the rest of the recordings from other stations. For each broadcast call, we then recorded its presence to compare this to human recorded data. Three of the recorders NE3, NE4, and Ex2 did not work, despite previous testing, and so data from these recorders was not available for analyses. We replicated the data from recorders Ex3, Ex4, and NE2 to match detection with people at those stations who were under the recorders that did not work (i.e., NE3, NE4, and Ex2).

#### 2.5.2 Data from human observers

Data were initially matched with the expected sequences broadcasted using the identification, direction, distance, and time recorded by observers, where this was provided. Despite the instructions, some observers did not write any information about time, distance, or direction. In these cases, we used the ruru and brown kiwi calls to decide at what point in the sequence each call went, together with data from the other person at the station and the annotated data from the autonomous recorders. This last one was only used as a last resort as a guide to decide whether a sound may have been heard. Data were scored as binary variables based on whether individual observers detected or failed to detect individual broadcast calls and whether they successfully identified the species. One of the human observers’ information was not used in the analyses because this individual did not follow any of the instructions, and his data were not comparable to that of the other human observers.

#### 2.5.3 Distances between the stations and speakers

GPS coordinates taken on site using a Garmin Rino and calibrated against map features were used to compute distance and direction using the calculator at http://www.movable-type.co.uk/scripts/latlong.html; this uses the *Haversine* formula to calculate the shortest distance over the earth's surface between points, giving an *as‐the‐crow‐flies* distance between the points:

$$a=\sin^{2}\left(\Delta \varphi /2\right)+\cos\varphi_{1}\cdot \cos\varphi_{2}\cdot \sin^{2}\left(\Delta \lambda /2\right).$$

$$c=2\cdot a\tan2\left(\sqrt{a},\;\sqrt{\left(1-a\right)}\right)$$

*Haversine* formula: d = R ⋅ c.

where φ is latitude, *λ* is longitude, and R is earth's radius (mean radius = 6,371 km).

#### 2.5.4 Altitude

We used the Google Earth Pro “show elevation profile” feature to obtain the altitude of each listening station and broadcasting site, and calculated the relative altitude or altitude difference between the recorder and the speaker (recorder altitude‐speaker altitude). Line of sight was deemed to have occurred when the broadcasting site was in direct line from the listening station without any geographical feature separating them.

#### 2.5.5 Broadcast direction in relation to listening station

The direction of the calls broadcasted in relation to the listening stations was calculated by measuring the angle between the two on a map in degrees, and giving a location (cardinal point) for the listening stations in relation to the broadcasting site (North, East, West, or South).

### 2.6 Statistical analyses

We considered each individual bird call broadcast as a trial and treated the data as a series of Bernoulli trials, with the success (1) or failure (0) of detection of that call as the binary variable. For human data, we used the presence of a bird in their survey sheet as a success and the lack of a bird as a failure. For the ARUs, we reviewed the recording both visually as a spectrogram in AviaNZ, and aurally through headphones, because some recorded calls were audible but not visible, to establish the success or failure of detection.

We then constructed a hierarchical generalized linear model by assembling the sequence of success (=1)/failure (=0) observations *y_i_* into a data vector. Each *y_i_* was represented by a random variable with a Bernoulli distribution *p_i_* (Equation 1) based on a sigmoid function (Equation 2), where esp*_i_* is a linear expression of covariate factors that we aimed to fit:

(1) $$y_{i}dbern\left(p_{i}\right)$$

(2) $$p_{i}\leftarrow \frac{1}{1+e^{-{\mathrm{esp}}_{i}}}$$

(3) $${\mathrm{esp}}_{i}\leftarrow \mathrm{int}+\beta_{r_{i}st_{i}}^{\mathrm{st}}+\beta_{r_{i}t_{i}}^{t}+\beta_{r_{i}}^{\mathrm{dis}}*{\mathrm{dis}}_{i}+\beta_{r_{i}}^{a}*a_{i}+\beta_{r_{i}l_{i}}^{l}+\beta_{r_{i}ca_{i}}^{\mathrm{ca}}+\beta_{r_{i}{\mathrm{dir}}_{i}}^{\mathrm{dir}}+\varepsilon_{i}$$

The majority of the terms in ${\mathrm{esp}}_{i}$ (Equation 3) were hierarchically modeled as normally distributed with zero mean and very small precision. The exceptions were the terms for the individual ARU/human observers. This covariate, for the human observers ($\beta_{1:13}^{r}$), was hierarchically modeled with its own linear model that accounts for previous experience, age and gender:

(4) $$\beta_{\forall i\in \left\{1:13\right\}}^{r}\mathrm{dnorm}\left(\mu_{i},0.0001\right)$$

(5) $${\underset{\leftarrow }{\mu }}_{i}\mathrm{int}p+\beta_{{\mathrm{vote}}_{i}}^{\mathrm{vote}}+\beta_{{\mathrm{fmc}}_{i}}^{\mathrm{fmc}}+\beta_{{\mathrm{kcs}}_{i}}^{\mathrm{kcs}}+\beta_{{\mathrm{os}}_{i}}^{\mathrm{os}}+\beta_{n_{i}}^{\mathrm{gen}}+\beta^{\mathrm{age}}*{\mathrm{age}}_{i}$$

Each person's individual contribution covariate was modeled as normally distributed around their $\mu_{i}$ (4).

The terms in Equation (5) are as follows:

$\beta_{vote_{i}}^{\mathrm{vote}}$ represents each person's self‐assessment of previous knowledge. The $\beta_{{\mathrm{fmc}}_{i}}^{\mathrm{fmc}}$ covariates account for previous experience in “five‐minute bird counts.” The $\beta_{{\mathrm{kcs}}_{i}}^{\mathrm{kcs}}$ covariates represent how many “kiwi call surveys” the person has attended. The $\beta_{{\mathrm{os}}_{i}}^{\mathrm{os}}$ covariates represent how many other surveys the person has previously attended (Table 2). All of these factors are proxies for experience, and hence, significant proportional differences within each group would be interpreted as the contribution of the person's experience to her/his ability in detecting a bird call.

The $\beta^{\mathrm{gen}}$ group is composed of only two classes and represents the observer's gender; a significant difference between the female and male covariate would be interpreted as a gender specific contribution to the person's ability in detecting a call. Finally, the $\beta^{\mathrm{age}}$ represents the influence of age on the ability of detecting calls. It is multiplied by the standardized ($z=\frac{x-\mu }{\sigma }$) age of each observer (Table 2).

For the ARU terms, $\beta_{14:24}^{r}$ was still hierarchically modeled, but without its own linear model, since there were no known individual differences that we were testing between the recording units. Thus, the $\left(\beta_{14:24}^{r}\right)$ were normally distributed with a normally distributed *sample mean* and very small precision. The *sample mean* in turn had a mean equal to zero and a very small precision.

In the full model:

The station covariates matrix, $\beta_{r_{i}{\mathrm{st}}_{i}}^{\mathrm{st}}$, represents the effect of the different stations on the persons’ detecting probability. They are included for completeness, since the ARUs are stationary, fixed at a station, these covariates are set to 0.

The trials covariates matrix, $\beta_{r_{i}t_{i}}^{t}$, represent the possible influence of time on people and ARUs (either people getting better with practice, or getting bored), or ARUs losing battery or failing, being composed of seven classes, one for each trial.

The $\beta_{r_{i}}^{r\mathrm{dis}}$ covariates represent the effect of the physical distance between the human observer/ARU and the broadcasting speaker; they are multiplied by the standardized ($z=\frac{x-\mu }{\sigma }$) distance of each detection event. Similarly, the $\beta_{r_{i}}^{a}$, intended to account for the effect of altitude difference on each person/ARU and are multiplied for the standardized ($z=\frac{x-\mu }{\sigma }$) altitude differences. The $\beta_{r_{i}l_{i}}^{l}$covariates represent the effect of being in/out of line of sight with the source of the broadcast call on each human observer/ARU. They are an array of pairs of mutually exclusive covariates at each detection event.

The $\beta_{r_{i}{\mathrm{dir}}_{i}}^{\mathrm{dir}}$ matrix of covariates is intended to account for the effect of a call coming from a certain direction on each person/ARU detection probability. It is structured in a sectorial fashion, with 8 covariates covering the 360 possible degrees from whence a call could be coming, 45° at a time (e.g., a call coming from the E‐NE sector ~70° would be in the second class, whereas one coming from the S‐SW sector ~200° would be in the fifth one); a significant (positively or negatively) value on any of these covariates would be interpreted as a person/ARU being more/less able to detect a call that comes from a certain direction. Because the broadcast were all toward the North, calls coming from the South would be in direct line with the ARUs/human observers and our expectation is that this direction would have a higher detection probability.

The $\beta_{r_{i}{\mathrm{ca}}_{i}}^{\mathrm{ca}}$ matrix of covariates represents the effect of each of the five different calls broadcasted on the detection probability of each human observer/ARU.

Lastly, the $\varepsilon_{i}$ covariates are overdispersion parameters, intended to account for unaccounted variability; significant values for this parameter would mean poor representation of the data variability by the other covariates.

After 70,000 burn‐in iterations, seven independent chains ran through JAGS (Plummer, 2016) in the R environment (R core team, 2018) using the *coda.samples()* command for 200,000 Markov chain Monte Carlo (MCMC) iterations with a thinning interval of 20 (i.e., retaining one value every twenty simulated steps for each variable) for a total of 70,000 assumed independent observations.

We ran the *effectiveSize()* command from the coda package to check the actual number of independent samples from the posterior probability densities. Subsequently, we randomized a matrix of indexes with 10 columns by 10% of the dataset size (4,860 observation = 486) rows, and sequentially removed 10% of the data points at a time to cross‐validate the model by checking the percentage of data points that were correctly estimated when the entry was deleted.

## 3 RESULTS

After the posterior sampling, the chain mixing was visually inspected and overall showed good mixing. The model described the data well; tenfold cross‐validation showed that the methods correctly accounted for 82.366% of the data points. Although at least one variable had an extremely small effective sampling size, meaning a high level of autocorrelation for some variables, most showed independent sampling (Minimum = 28.21; 1st Quantile = 60,195.35; Median = 67,734.89; Mean = 60,562.09; 3rd Quantile = 70,000.00; Maximum = 73,613.36). Since most of the covariates were modeled as being zero mean with very small precision, we can consider those with high‐density intervals (posterior probability density between the 1st and 5th quantile) completely above or below zero as significantly affecting the detection probability throughout the analysis.

**Figure 3:**
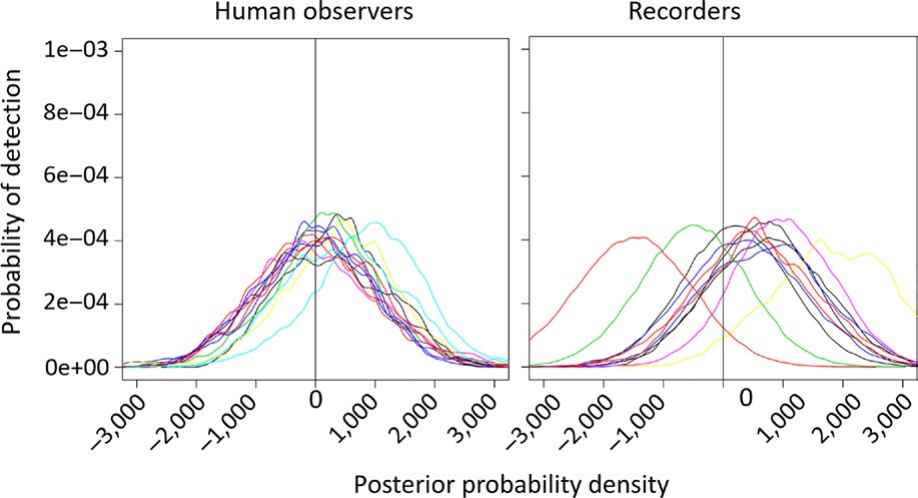
The individual influence of each ARU or human observer on detection probability of broadcast sounds at the Rawhiti Experiment. Each person and each recorder corresponds to a different color line in the plots. This posterior probability density plot represents the distribution of the *individual contribution* covariate after the MCMC runs (most of their prior distributions were modeled as normally distributed with zero mean and very small precision). The vertical line, placed on 0, is there to help visualize the proportion of each covariate's posterior that is above or below this point. Covariates with posterior distributions completely above or below zero have more consistent effects on the detection probability

**Figure 4:**
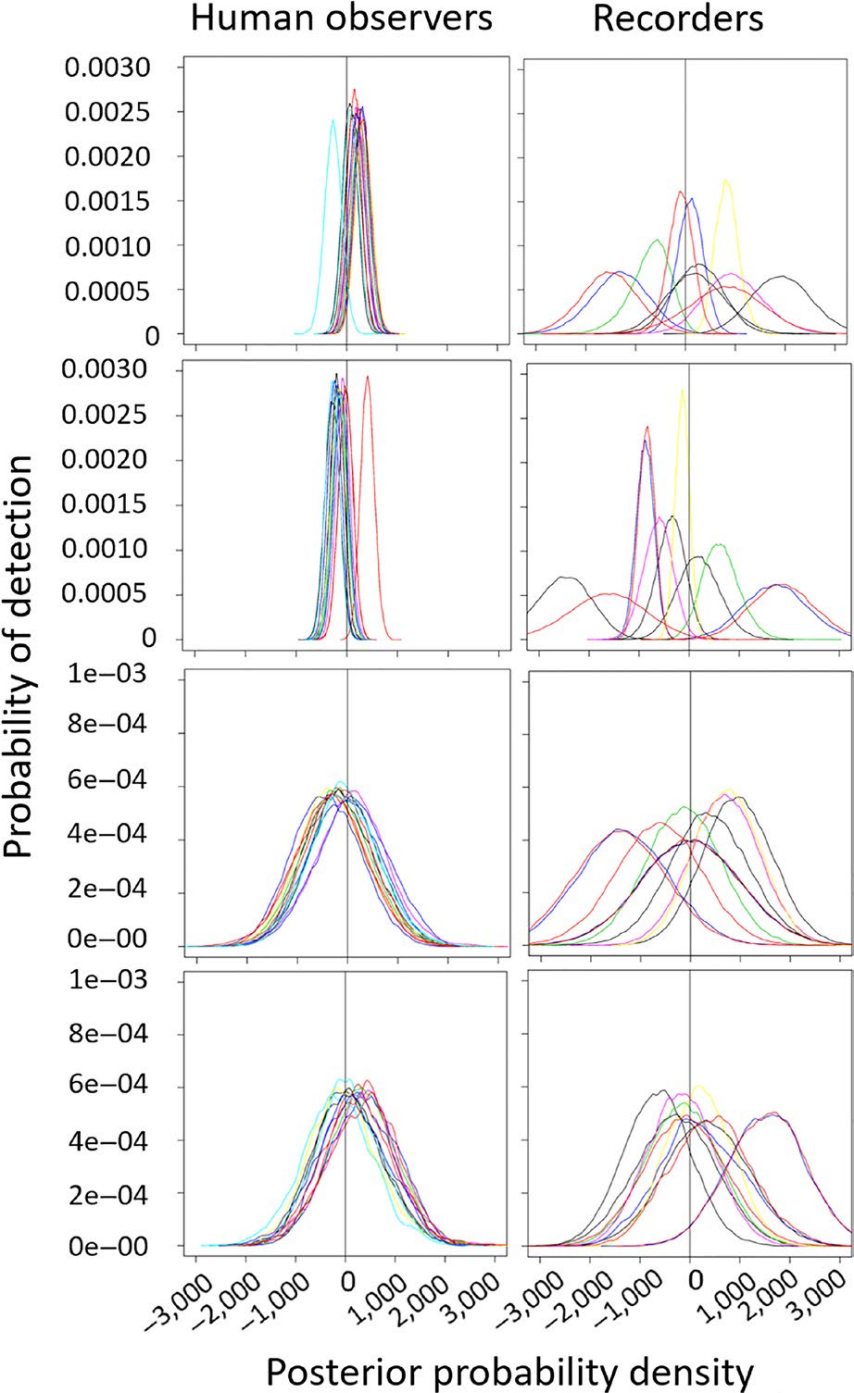
Influence of line of sight (LOS), out of sight (OOS), altitude (AL), and distance (DIS) covariates on the detection probability of ARUs and human observers to broadcast calls during the Rawhiti Experiment. Each person and each recorder corresponds to a different color line in the plots. These posterior probability density plots represent the distribution of each of the covariates after the MCMC runs (most of their prior distributions were modeled as normally distributed with zero mean and very small precision). The vertical line, placed on 0, is there to help visualize the proportion of each covariate's posterior that is above or below this point. Covariates with posterior distributions completely above or below zero have more consistent effects on the detection probability

**Figure 5:**
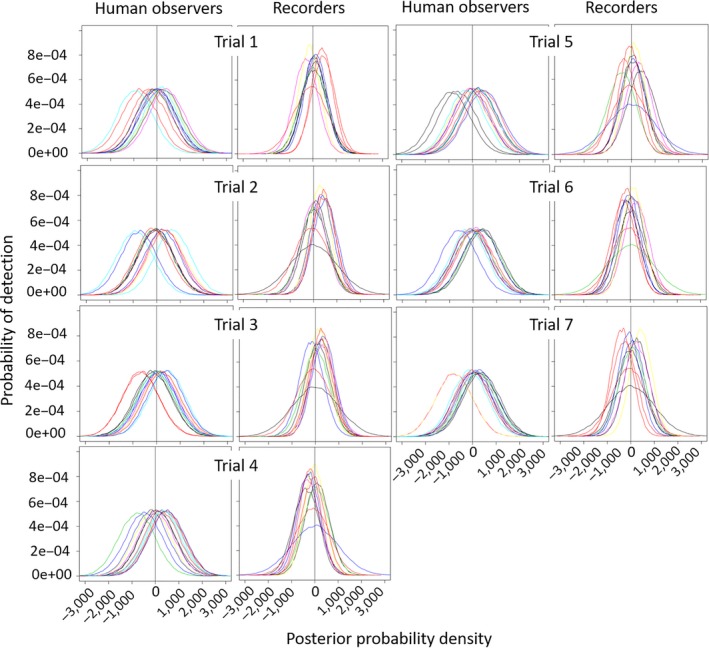
Influence of trial on the detection probability of ARUs and people to broadcast calls during the Rawhiti Experiment. Each person and each recorder corresponds to a different color line in the plots. This posterior probability density plot represents the distribution of the trial covariate after the MCMC runs (most of their prior distributions were modeled as normally distributed with zero mean and very small precision). The vertical line, placed on 0, is there to help visualize the proportion of each covariate's posterior that is above or below this point. Covariates with posterior distributions completely above or below zero have more consistent effects on the detection probability

**Figure 6:**
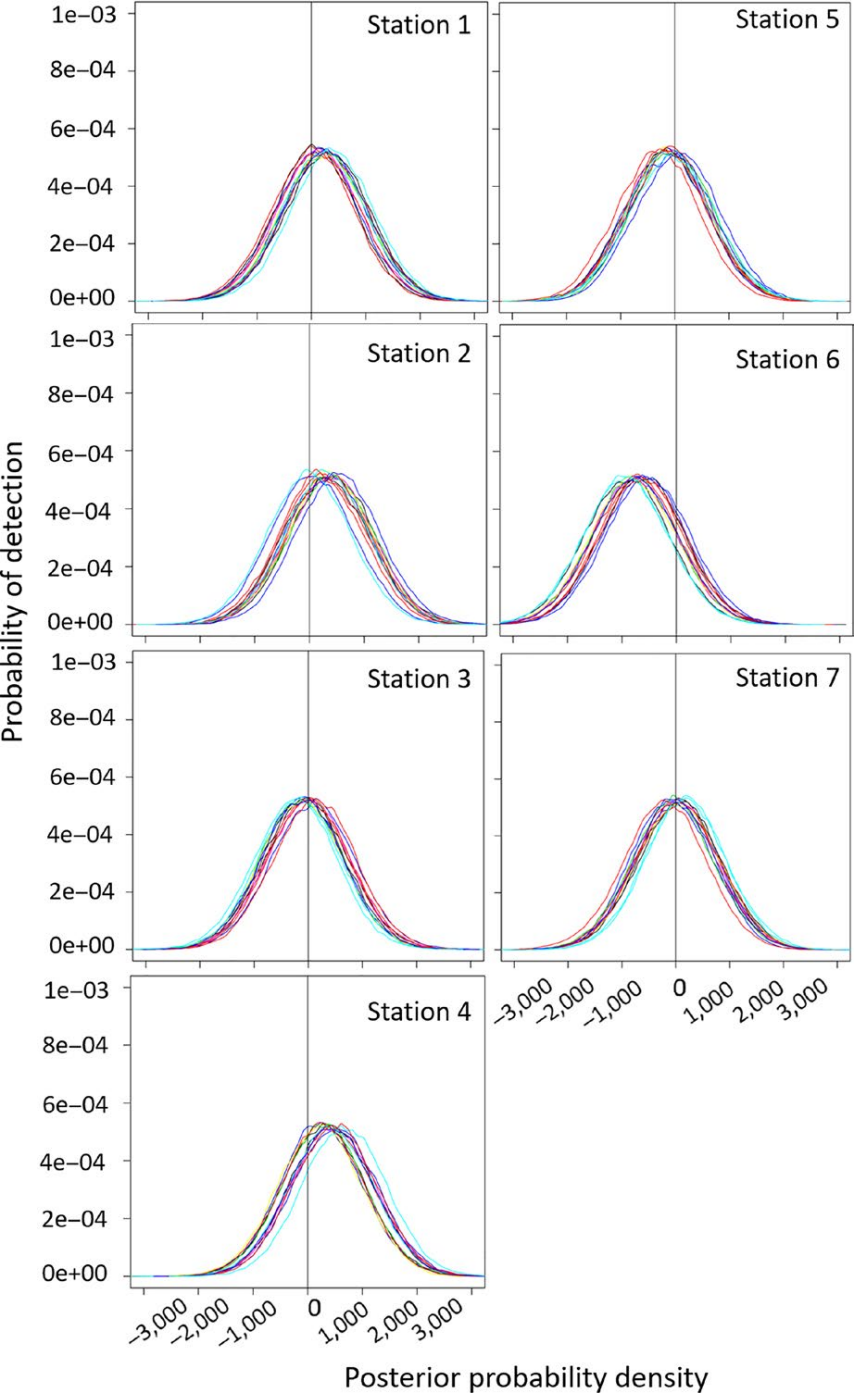
Influence of station covariate on the detection probability of human observers to broadcast calls during the Rawhiti Experiment. Each human observer corresponds to a different color line in the plots. This posterior probability density plot represents the distribution of the station covariate after the MCMC runs (most of their prior distributions were modeled as normally distributed with zero mean and very small precision). The vertical line, placed on 0, is there to help visualize the proportion of each covariate's posterior that is above or below this point. Covariates with posterior distributions completely above or below zero have more consistent effects on the detection probability

**Figure 7:**
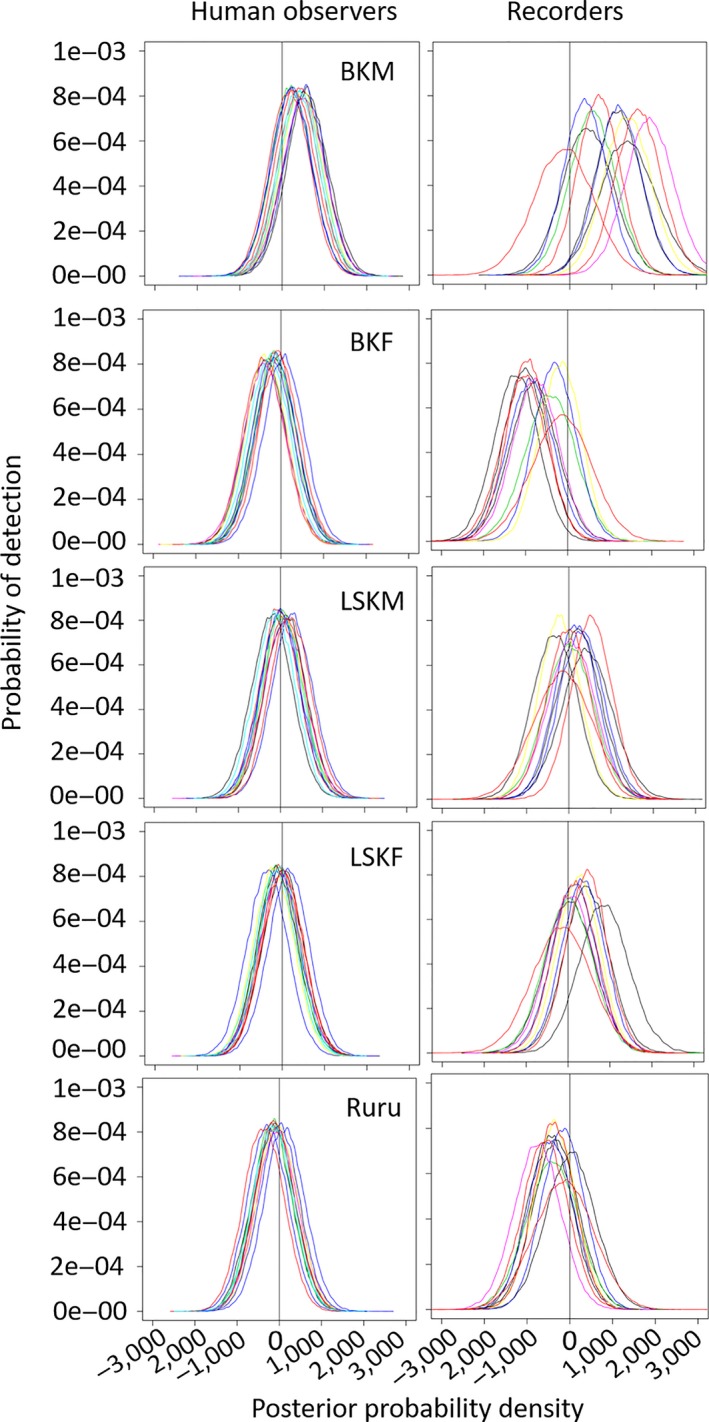
Influence of species‐call broadcast on the detection probability of ARUs and people to those calls during the Rawhiti Experiment. BKF: brown kiwi female; BKM: brown kiwi male; LSKF: little spotted kiwi female; LSKM: little spotted kiwi male; RR: ruru. Each person and each recorder corresponds to a different color line in the plots. These posterior probability density plots represent the distribution of each species‐call covariate after the MCMC runs (most of their prior distributions were modeled as normally distributed with zero mean and very small precision). The vertical line, placed on 0, is there to help visualize the proportion of each covariate's posterior that is above or below this point. Covariates with posterior distributions completely above or below zero have more consistent effects on the detection probability

**Figure 8:**
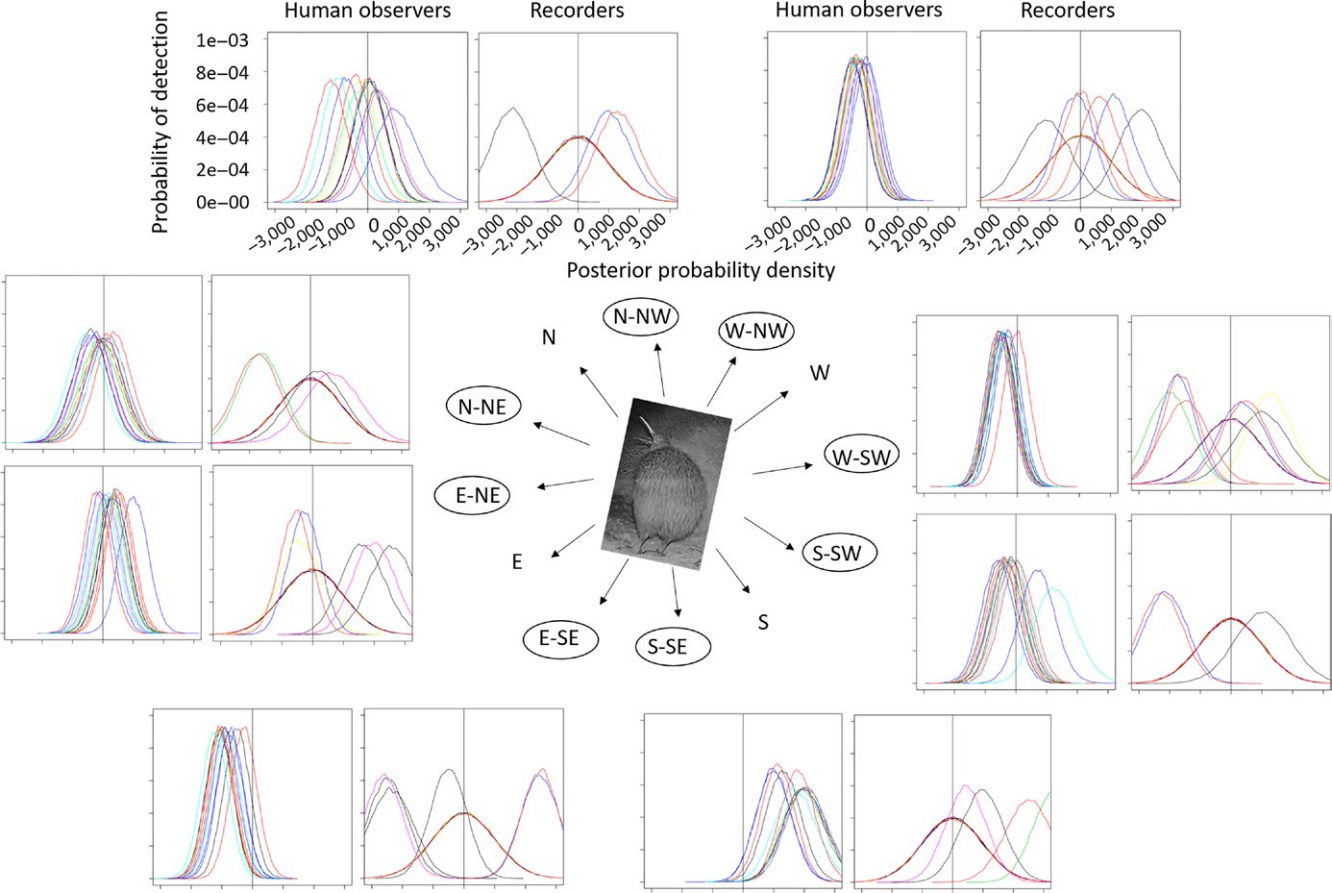
Influence of broadcast direction on the detection probability of ARUs and human observers to broadcast calls during the Rawhiti Experiment. Axis labels are demonstrated in the top plots. All calls were broadcast to the North. The plots show probability of detection by human observers and recorders located at the encircled positions in relation to the broadcast. Each person and each recorder corresponds to a different color line in the plots. These posterior probability density plots represent the distribution of each direction covariate after the MCMC runs (most of their prior distributions were modeled as normally distributed with zero mean and very small precision). The vertical line, placed on 0, is there to help visualize the proportion of each covariate's posterior that is above or below this point. Covariates with posterior distributions completely above or below zero have more consistent effects on the detection probability. E: East; N: North; S: South; W: West

There was inter‐ and intravariation in overall detection probability between people and ARUs as illustrated in Figures 3, 4, 5, 6, 7, 8. The variation was larger among ARUs than people. ARUs however performed significantly better than human observers, recording 1,631 (60%; of those, 1,546 (57%) were visible on the spectrograms, the others were detected by listening) of the 2,731 broadcast calls versus 1,434 (53%) for humans. When we look at the effect of each ARU and human individual contribution on detection probability (Figure 3), ARUs had overall significantly more variable individual contribution, probably due to their position. The high level of variation in the effect of distance and altitude on the ARUs supports this proposition. For humans, the individual contribution contained the information about experience, and as expected, this did not influence their ability to hear and record a sound. The next step is to find out if their experience affected their ability in distinguishing the calls of each species when broadcast under the different circumstances of the experiment (in preparation).

Overall, altitude, line of sight, and distance had a much stronger effect on ARUs than people (Figure 4). The relative altitude between stations and speakers varied from −21 to 30 m (Table 6). Differences in altitude between the source of the call and the human observer/ARU had a much stronger effect on the detection probabilities of ARUs than human observers, with sounds broadcast from speakers at similar altitude to ARUs having better detection probability. Distances from speaker to stations varied from 25 to 314.4 m and affected both ARUs and humans. The greater the distance between speaker and ARU or human observer, the lower the detection probability (Figure 4).

**Table 6:** Distances between stations and broadcast and altitudinal differences between stations and broadcast (=recorder altitude‐speaker altitude; therefore, a positive value indicates that the speaker (bird) is lower than the recorder and vice versa)

	Speaker 1	Speaker 2	Speaker 3	Speaker 4	Speaker 5	Speaker 6
Station	Distance					
1	84.6	136.7	267.5	30.4	167.9	264.4
2	55.8	184.3	314.4	115	193	281.3
3	61.5	78.2	209.1	80.2	95.9	191.5
4	113.5	57.3	154.1	149.7	25.3	116.7
5	172.5	51	90.8	176.9	41.7	93.5
6	235.8	112.8	36.1	237.8	98.7	76.2
7	260.6	158.2	83.8	283.5	124.7	43
	Relative altitude					
1	22	−2	3	−2	−4	1
2	5	−19	−14	−19	−21	−16
3	16	−8	−3	−8	−10	−5
4	25	1	6	1	−1	4
5	30	6	11	6	4	9
6	17	−7	−2	−7	−9	−4
7	27	3	8	3	1	6

There was higher degree of variation in detection probability for humans than ARUs during the various trials (Figure 5). Generalizing, human observers varied in their performance much more markedly than ARUs. For both human and ARUs, the specific location or individual differences had an effect on detection. For example, all human observers had significantly lower detection probability when at station 6 (Figure 6); in trial 7 (Figure 5), one of the ARUs at station 6 (recorder a; represented by the red line) had high detection of sounds, while the other (recorder b; represented by the yellow line) had low detection.

Station had a major influence on the detection probability of human observers (but not ARUs, which did not move during the experiment) with human observers having significantly lower detection probability when listening at station 6, and relatively higher at stations 1, 2, and 4 (Figure 6).

There was a bias in the ARUs detection probability of some of the broadcast calls together with high variation in detection probability between ARUs (Figure 7). In general, ARUs had significantly lower detection probability for brown kiwi female (BKF) calls, and a higher detection probability for brown kiwi male (BKM) calls (Figure 7). Ruru calls were also less likely to be recorded by ARUs. People had similar detection probabilities for all calls (Figure 7).

Direction of the broadcast was not expected to have a clear effect on the detection probability; however, it demonstrated a big influence, as illustrated in the variety of detection probabilities in Figure 8. We could have expected some particular directions to have an effect on the ARUs detection probability (since they are fixed in a location), but from the figure it seems that differences between individual recorders and people are more important than the direction of the broadcast. Note that human observers (and ARUs, but these were stationary) were better at hearing calls coming from specific directions. For example, human observers 1 and 2 had difficulties hearing sounds coming from the W‐SW regardless of the station they were listening from.

## 4 DISCUSSION

Overall, we found that human observers and recorders were similar in the detection of sounds supporting some of the non‐experimental studies (Table 1), although the variables we measured affected them differently. These differences may account for disagreements between studies. Human observers were relatively homogeneous in their detection probability, with very little variability between individuals; this is despite wide differences in age and experience between human observers. In contrast, ARUs had more variability in detection probability, with some ARUs having detection probabilities significantly higher than any of the human observers in the study and some significantly lower. The individual contribution of each human observer to detection probability was also less variable than that of recorders. It is possible that less homogeneity of the ARUs resulted from the fact that the ARUs are highly susceptible to the surrounding objects in the environment, for example, different forest densities and obstacles.

Distance affected ARUs detection probability more than humans, with calls broadcast farther away generally having a lower detection probability, as we hypothesized. ARUs have been found to have a smaller hearing radius than humans do (Yip, Leston, Bayne, Solymos, & Grover, 2017), and this probably explains the greater effect of distance on ARUs found in this study. Other differences and inconsistencies in this relationship are probably due to (a) the location of the station in relation to the speaker as is suggested by the strong influence of station on human observers’ detection probability; (b) the exact location of the ARU, as ARUs in the same area but a small distance apart had significantly different detection probabilities; and (c) human's directional filtering ability, which allows them to move their head in the direction of the sound.

To our knowledge, no other study has examined the effect of relative altitude between bird and recorder, and within the landscape (valley vs. hilltop) in the detection probability of humans and ARUs. In New Zealand, this is of special importance, as survey stations aimed at detecting kiwi are located at hilltops, assuming that this improves detection. Our results suggest that generally speaking, birds calling from hillsides and those relatively higher or lower from recording sites are less likely to be detected by ARUs, and to a lesser extent by human observers, than those at a similar altitude to listening stations. ARUs had better detection probability if broadcast was line of sight of the location of the ARU. These differences between ARUs and humans are probably due to the immobility of the ARUs and human's directional filtering ability. As well as being able to move their heads, humans locate sound sources (above, below, front, and back) using different stimulus cues, such as interaural level difference, interaural time difference, and spectral cues, something ARUS cannot do. Humans were strongly affected by Trial, but this seems to be the result of the strong influence of station 6 on human observers’ detection probability. Station 6 was located in a deep valley close to a small stream. Both humans and ARUs had difficulties detecting calls from this station. The sound of the stream was not enough to prevent ARUs and humans from recording the broadcast calls, so the depth of the valley was probably the feature that prevented sound reaching ARUs/humans. Overall, we conclude that listening stations would have better detection probability if located like station 4, in a hill overlooking and central to an area to be surveyed.

ARUs exhibited a recording frequency bias: Relatively lower frequency female brown kiwi and ruru *trill* and *weow* calls had lower detection probabilities. Yip et al. (2017) also found differences between the frequencies recorders detected when comparing a range of ARUs; some recorders were more attuned to higher frequencies and vice versa. These authors argue that differences in detectability due to sound's frequencies will affect the distance at which recorders can detect sounds and of course comparability between human and ARU surveys. Our results support these conclusions and indicate that any calibrations will have to be not only ARU brand specific but also consider individual ARUs. Further, differences between ARUs at the same stations suggest that the exact location of the device is important in terms of what they can record, and that consideration should be given to this when selecting the recorder location and also having more than one device per listening station or more than one microphone per ARU. While some commercially available ARUs have two microphones, many have a single omnidirectional microphone. Having more than one microphone could also be used to enable the estimation of location/direction of the sounds by the ARUs, one of the most important criticisms of ARUs (Table 1).

In this experiment, we measured the overall detection probability, the individual contribution of each human observer and ARU to detection probability, and compared the effect of distance, relative altitude, location, species call, and trial on the detection probability of ARUs and humans. We found that human detection probability is more uniform between observers (despite big differences in age and experience of observers) than ARUs’, but ARUs can have higher detection probabilities if positioned properly. The variables measured acted differently on ARUs and human observers. We think that the next step is to measure the effect of these variables on human identification capability as well as their effect on the data quality of ARUs, particular with respect to precise location of ARUs. This information is needed to understand human errors in surveys as well as to allow proper calibration between human surveys and ARU surveys, and to inform software production for the automatic identification of species.

## CONFLICT OF INTEREST

None declared.

## **AUTHOR** **CONTRIBUTIONS**

Conceived and designed: IC and SM with contribution from NP. Song preparation: NP and IC. Logistics: IC; Field work: IC and SM. Data extraction and preparation: LB, NP, and IC. Statistics: ADR and IC. Manuscript preparation: IC with comments from all other authors.

### 1 Data Accessibility

Dryad https://doi.org/10.5061/dryad.cb43g0n.

Data files: analyses code, Raw data, Hot‐map figures.

Sound files: Brown kiwi male, Little spotted kiwi female, Little spotted kiwi male, Ruru trill‐weow, Brown kiwi duet, Brown kiwi female, Broadcast sequence.

## APPENDIX 1

### Data sheets used by observers during an experiment testing their detection and identification skills.

**Table:**

Station	Time start (h:m:s)	Time finish (m:s)	Bird species	Bird sex (M, F, Duet)	Compass reading (°)	Estimated distance (m)
						
						
						
						
						
						
						
						
						
						
						
						
						
						
						
						
						
						
						
						
						
						
						
						
						
						
						

## APPENDIX 2

### Survey sheet completed by human observers in the Rawhiti Experiment to give an indication of their level of expertise.

Date: _______________

Experimental site: _______________

Observer's name: _______________

Age: _______________

Gender: _______________

Expertise (circle one answer) knows:

1. Most New Zealand species song well
2. Most forest birds’ song including rare species, i.e., kiwi hihi, tieke
3. A variety of common species song, i.e., riroriro, fantail, silvereye, tui, ruru
4. Only common species

Have you participated in?

1. 5MBC—number of years _______________
2. Kiwi call surveys—number of years __________________
3. Other types of birds surveys—number of years _____________
